# Association of non-shockable initial rhythm and psychotropic medication in sudden cardiac arrest

**DOI:** 10.1016/j.ijcha.2020.100518

**Published:** 2020-04-22

**Authors:** Janna P. Kauppila, Antti Hantula, Lasse Pakanen, Juha S. Perkiömäki, Matti Martikainen, Heikki V. Huikuri, M. Juhani Junttila

**Affiliations:** a Research Unit of Internal Medicine, Medical Research Center Oulu, Oulu University Hospital and University of Oulu, Finland; b Forensic Medicine Unit, National Institute for Health and Welfare, and Department of Forensic Medicine, Research Unit of Internal Medicine, Medical Research Center Oulu, University of Oulu, Oulu, Finland; c Center for Pre-hospital Emergency Care, Oulu University Hospital, Oulu, Finland

**Keywords:** Asystole, Medico-legal autopsy, Psychotropic medication, Pulseless electrical activity, Sudden cardiac arrest

## Abstract

**Background:**

Asystole (ASY) and pulseless electrical activity (PEA) have a poor outcome during sudden cardiac arrest (SCA). Psychotropic medication has been associated with a risk for sudden cardiac death (SCD). Our aim was to study the association of psychotropic medication with ASY/PEA during SCA.

**Methods and results:**

A total of 659 SCA subjects were derived from the emergency data of Oulu University Hospital (2007–2012). Subjects with non-cardiac origin of SCA and over 30-minute delay to rhythm recording were excluded. Population included 222 subjects after exclusions (mean age 64 ± 14 years, 78% males). Initial rhythm was ventricular fibrillation (VF) or ventricular tachycardia (VT) in 123 (55%), ASY in 67 (30%) and PEA in 32 (14%) subjects. The delay (collapse to rhythm recording) was similar in VF/VT and ASY/PEA subjects (median 8 min [1st–3rd quartile 3–12 min] versus 10 [0–14] minutes, p = 0.780). Among VF/VT subjects underlying cardiac disease was more often ischemic compared to ASY/PEA subjects (85% versus 68%, p = 0.003). Psychotropic medication was associated with ASY/PEA rhythm (OR 3.18, 95%CI 1.40–7.23, p = 0.006) after adjustment for gender, age and underlying cardiac disease. Subsequently, antipsychotics (OR 4.27, 95%CI 1.28–14.25, p = 0.018) were more common in the ASY/PEA group. Benzodiazepines and antidepressants were not associated with ASY/PEA.

**Conclusion:**

Psychotropic medication and especially antipsychotics are associated with non-shockable rhythm during SCA and may lower the possibility of survival from the event. This might partly explain the risk of SCD related to psychotropic medication.

## 1 Introduction

It is estimated that from 4 to 5 million sudden cardiac deaths (SCD) occur in the world annually [1]. The initial electrocardiographic rhythm recorded after collapse is a key factor in survival from sudden cardiac arrest (SCA). Ventricular fibrillation (VF) and ventricular tachycardia (VT) can often be successfully converted by defibrillation shock to a rhythm which sustains circulation. They are therefore also called shockable rhythms. Partly due to the effective therapy, shockable rhythms have been described to have a higher survival rate (10–46%) compared to asystole (ASY) and pulseless electrical activity (PEA) (1–16 % combined), which are referred to as non-shockable rhythms [2], [3], [4], [5], [6]. According to recent studies, proportion of VF/VT seems to be decreasing and ASY and PEA increasing as initial rhythms during SCA [1], [4], [5], [7], [8]. For decades, less efforts have been made to study the determinants of ASY and PEA. In previous studies, use of psychotropic medication and especially antipsychotics has been associated with an increased risk of SCD [9], [10], [11]. The mechanisms of this association are yet somewhat unclear. The effect of psychotropic medication on the initial rhythm during SCA might explain this association at least in part. The Oregon Sudden Unexpected Death Study demonstrated that use of psychotropic drugs increases the risk of PEA as the initial rhythm opposed to VF/VT at the time of SCA [7]. However, to our knowledge, the association between use of psychotropic drugs and ASY has not yet been examined. Our aim was to study the association of psychotropic medication with non-shockable initial rhythm at the time of SCA. In this study we examined the use of psychotropic medication among SCA subjects with VF/VT, ASY or PEA as the initial presenting rhythm.

## 2 Methods

**Fig. 1:**
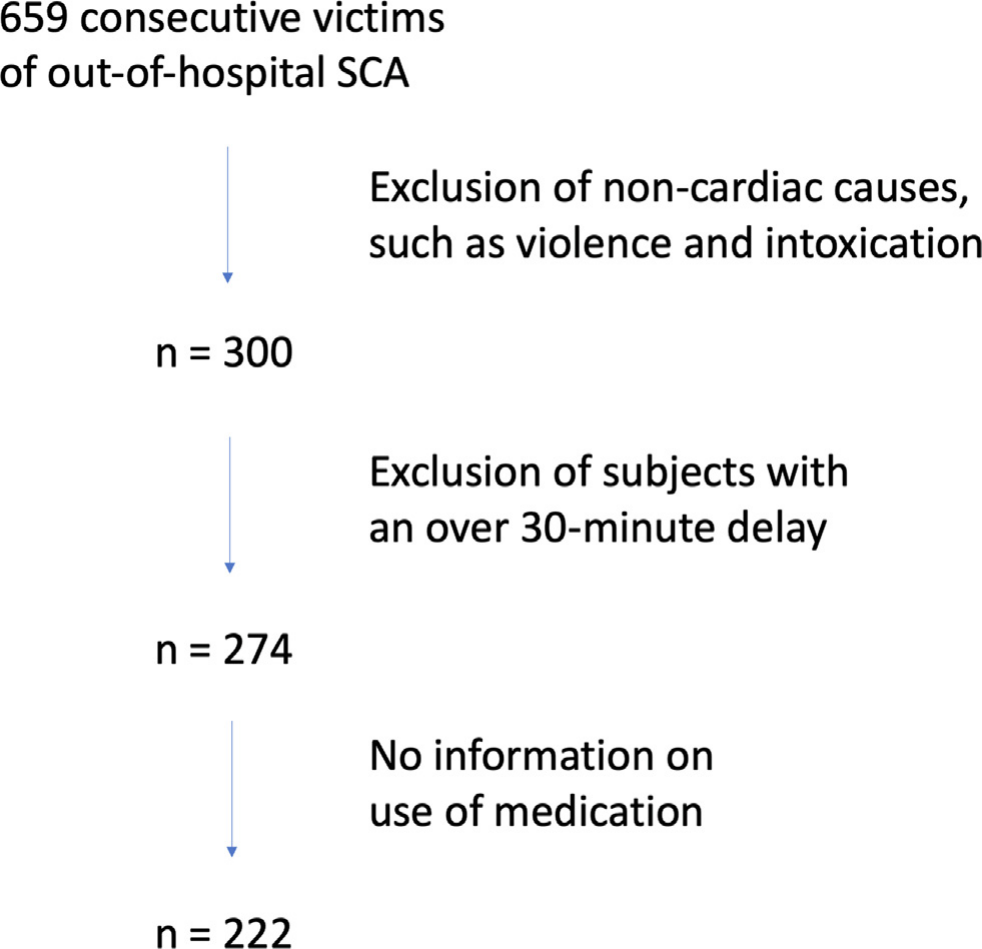
Selection of the study population.

Study population was derived retrospectively from the emergency service data of Oulu University Hospital (Oulu, Finland) area. The emergency medical service system serves a population of approximately 741 000 and is equipped with ambulances, a medical helicopter and a mobile unit with an emergency physician on board. The data included 659 consecutive out-of-hospital cardiac arrest patients with a documented rhythm at the time of SCA during years 2007–2012. The rhythm was recorded with electrocardiography by emergency personnel at the scene. Utstein recommendation for uniform reporting of cardiac arrest [12] was used in documenting the emergency service data. This data included the initial rhythm, delay from collapse to recording of rhythm and patient outcome. Non-cardiac causes, such as acts of violence and clear cases of trauma, suicide and intoxication (n = 359), as well as cases with an over 30-minute delay from collapse to recording of rhythm (n = 26), and cases with no information on use of psychotropic medication during the 2-year-period prior to the incident (n = 52) were excluded. The selection of the population is illustrated in Fig. 1. In this study, SCA is defined as a witnessed unexpected pulseless condition of cardiac cause.

A total of 222 (mean age 64 ± 14 years, 78% males) cases were included in the study, of which 116 subjects were victims of SCD and thus underwent medico-legal autopsy with predefined criteria [13] in order to exclude non-cardiac causes. All cases of SCD are also a part of the Finnish Genetic Study of Arrhythmic Events (Fingesture), which has been reported in detail before [13], [14], [15]. Medico-legal autopsies were performed at the Department of Forensic Medicine of the University of Oulu and National Institute for Health and Welfare, Oulu, Finland. Data on these SCD victims was gathered from autopsy reports, medical records and questionnaires to closest family members. All autopsies included histologic examination. Toxicology investigation, including post-mortem blood analysis for psychotropic medications in blood, was performed in cases with suspicion of toxic exposure. The concentrations of medications in post-mortem blood were on therapeutic levels in all cases, as all subjects with supratherapeutic levels of psychotropic medication in blood were excluded from the study.

Hundred and six participants were successfully resuscitated, and they survived to hospital admission. In these cases, SCA was confirmed to be due to a cardiac cause by clinical examination, including echocardiography and coronary angiography. Information on underlying diagnosed cardiac disease and prior medication was acquired from medical records in the electronic archives of Oulu University Hospital. No information was gained on further health conditions of the survivors, such as neurological function after the incident. Because of this, we divided the study population to those who died in spite of attempted resuscitation and those who survived to hospital admission.

The primary focus of this study was to assess the association between use of psychotropic medication and non-shockable rhythm (ASY/PEA) versus shockable rhythm (VF/VT). Psychotropic medications included in the study were benzodiazepines, antipsychotics and antidepressants. Information about use of metformin was used as a surrogate for a history of diabetes in the subjects.

The study complies with the declaration of Helsinki and was approved by the ethics committee of Northern Ostrobothnia Hospital District (Oulu University Hospital). National Institute for Health and Welfare and the Regional State Administrative Agency of Northern Finland approved the review of medico-legal autopsy data by the investigators.

Chi-square test was used to detect significant differences in the distribution of dichotomized variables. Gaussian distribution of variables was evaluated by skewness test. When comparing continuous variables of two groups, independent samples *t*-test was used for variables with normal distribution and Mann-Whitney test for variables with non-normal distribution. Binary logistic regression analysis was used to determine the odds ratio (OR) for risk of non-shockable rhythm for users of different psychotropic medication. The IBM Statistical Package for Social Studies 25 (SPSS Inc., Chicago, IL) was used to perform the analyses, and two-sided p-values < 0.05 were considered statistically significant.

## 3 Results

A total of 222 subjects (mean age 64 ± 14 years, 78% males) of SCA with a known initial rhythm were included in the analysis. Out of all subjects, 106 (48%) were successfully resuscitated. VF/VT was recorded as the initial rhythm in 123 (55%) subjects, ASY in 67 (30%) subjects and PEA in 32 (14%) subjects. The VF/VT group had more males compared to the non-shockable (ASY/PEA) group (Table 1). Subjects with VF/VT were significantly younger than subjects with non-shockable rhythm. The delay from collapse to recording of rhythm was similar in the two groups. There were no significant differences in body mass index, history of hypertension or diabetes between VF/VT and ASY/PEA groups. However, information about body mass index was achieved only in some of the victims of SCD, as we did not have information on body mass index of the survivors. Thus, the information was missing in a total of 150 (68%) subjects. The underlying cardiac disease was more frequently ischemic in the VF/VT group compared to the ASY/PEA group (Table 1). ASY/PEA rhythm was significantly more common in victims of SCD versus survivors of SCA (Table 2). The delay from collapse to recording of rhythm was longer in victims of SCD compared to survivors of SCA. There were no differences in age, gender, type of underlying cardiac disease or history of hypertension or diabetes between victims of SCD and survivors of SCA (Table 2).

**Table 1:** Characteristics of the study group with VF/VT compared to the study group with ASY/PEA.

	Total n = 222	VF/VT n = 123 (55%)	ASY/PEA n = 99 (45%)	P-value
Age, years (mean ± standard deviation)	64 ± 14	62 ± 13	67 ± 15	0.028
Gender, male	78%	84%	71%	0.020
Delay, minutes (median [1st–3rd quartile])	9 (1–13)	8 (3–12)	10 (0–14)	0.780
Underlying cardiac disease, ischemic	77%	85%	68%	0.003
BMI, kg/m^2^ (mean ± standard deviation)	29 ± 8	28 ± 5	30 ± 9	0.340
Diabetes	12%	12%	12%	0,987
Hypertension, %	41%	38%	49%	0.291

Abbreviations: ASY = asystole, BMI = body mass index, PEA = pulseless electrical activity, VF = ventricular fibrillation, VT = ventricular tachycardia.

**Table 2:** Initial rhythms and medications of victims of SCD versus survivors of SCA.

	Total (%) n = 222	Victims of SCD (%) (n = 116)	Survivors of SCA (%) (n = 106)	P-value
ASY/PEA as initial rhythm	99 (45)	77 (66)	22 (21)	<0.001
Age, years (mean ± standard deviation)	64 ± 14	64 ± 14	64 ± 15	0.833
Gender, male	78%	78%	78%	0.898
Delay, minutes (median [1st–3rd quartile])	9 (1–13)	10 (3–15)	7 (0–11)	0.020
Underlying cardiac disease, ischemic	77%	73%	81%	0.165
Diabetes	12%	12%	12%	0.965
Hypertension	41%	38%	43%	0.453
Psychotropic medication	36 (16)	27 (23)	9 (9)	0.003
Benzodiazepines	17 (8)	14 (12)	3 (3)	0.009
Antidepressants	20 (9)	15 (13)	5 (5)	0.031
Antipsychotics	16 (7)	13 (11)	3 (3)	0.016
Multiple types of psychotropic medication	15 (7)	13 (11)	2 (2)	0.006

Abbreviations: ASY = asystole, PEA = pulseless electrical activity, SCA = sudden cardiac arrest, SCD = sudden cardiac death.

Thirty-six (16%) subjects were users of psychotropic medication. Out of 36 users of psychotropic medication, 15 (42%) subjects were users of multiple types of psychotropic medication. Use of psychotropic medication was more common in victims of SCD. In a separate analysis of different types of psychotropic medication, use of benzodiazepines, antidepressants, antipsychotics, and multiple types of psychotropic medication was also more common in victims of SCD (Table 2). The use of medication in VF/VT and ASY/PEA groups is shown in Table 3.

**Table 3:** Use of psychotropic medication in subjects with VF/VT and ASY/PEA at the time of SCA.

	Total (%) n = 222	VF/VT (%) n = 123 (55)	ASY/PEA (%) n = 99 (45)
Psychotropic medication	36 (16)	10 (8)	26 (26)
Benzodiazepines	17 (8)	5 (4)	12 (12)
Antidepressants	20 (9)	6 (5)	14 (14)
Antipsychotics	16 (7)	4 (3)	12 (12)
Multiple types of psychotropic medication	15 (7)	5 (4)	10 (10)

Abbreviations: ASY = asystole, PEA = pulseless electrical activity, VF = ventricular fibrillation, VT = ventricular tachycardia.

Out of 116 victims of SCD, post-mortem blood analysis was performed in 43 (37%) cases. Out of these subjects, 15 (35%) had elevated psychotropic medication concentration in blood. The type of psychotropic medication found in blood analysis was benzodiazepines in 12 (28%) subjects, antidepressants in 6 (14%) subjects, and antipsychotics in 4 (9%) subjects. Eight (19%) subjects had multiple types of psychotropic medication in post-mortem blood. Out of the 43 subjects to whom post-mortem blood analysis was performed, 79% had the assumed medications in blood. This percentage includes the subjects with no assumed use of medication and no medication found in post-mortem blood. The concentrations of medications in post-mortem blood were on therapeutic levels in all cases.

In a logistic regression model of determinants of non-shockable rhythm versus shockable rhythm, use of psychotropic medication was independently associated with non-shockable rhythm, even after adjustment for gender, age and underlying cardiac disease (Table 4). In a separate analysis of different psychotropic medications, use of antipsychotics was associated with ASY/PEA rhythm after adjustment for gender, age and underlying cardiac disease. Use of benzodiazepines and antidepressants was associated with ASY/PEA rhythm, but the association was not significant after adjustments (Table 4).

**Table 4:** Medications as determinants of ASY/PEA versus VF/VT as the initial rhythm.

	Risk for ASY/PEA without adjustments OR (95%CI)	P-value	Risk for ASY/PEA with adjustments OR (95%CI)*	P-value
Psychotropic medication	4.02 (1.83–8.84)	0.001	3.18 (1.40–7.23)	0.006
Benzodiazepines	3.29 (1.12–9.69)	0.030	3.00 (0.96–9.35)	0.058
Antidepressants	3.25 (1.20–8.80)	0.020	2.11 (0.73–6.15)	0.170
Antipsychotics	4.10 (1.28–13.15)	0.018	4.27 (1.28–14.25)	0.018

Abbreviations: ASY = asystole, CI = confidence interval (*adjusted for gender, age and underlying cardiac disease), OR = odds ratio, PEA = pulseless electrical activity.

In a logistic regression model of determinants of death at the time of SCA, use of psychotropic medication was associated with death at the time of SCA (OR 3.27, 95%CI 1.46–7.33, p = 0.004), but the association was not significant after adjustment for non-shockable rhythm and delay from collapse to recording of rhythm (OR 1.83, 95%CI 0.74–4.53, p = 0.189).

## 4 Discussion

The results of this study allude to a significant influence of use of psychotropic medication, especially antipsychotics, on the risk of non-shockable rhythm at the time of SCA. In contrast to antipsychotics, use of antidepressants might not have a significant influence on the risk of non-shockable rhythm at the time of SCA.

Survival rates of out-of-hospital SCA seem to be improving, which is thought to be due to faster and more efficient responses by bystanders and emergency medical service [16]. Agarwal et al [4] reported a sufficiently high survival rate to hospital discharge among patients with VF at the time of SCA (46.3%) and the survival rate improved over the 18-year study period. Survival rates for non-shockable rhythms during SCA continue to be fairly poor. A large, population-based study by Andrew et al [3] demonstrated that patients with PEA have a better survival rate to hospital discharge compared to ASY (5.9% versus 1.1%, p < 0.001) with no significant improvement observed over the 10-year study period. Bergström et al [5] reported significantly higher survival rates among patients with PEA compared to ASY (16.5% versus 10.6%, p < 0.0001) and increasing survival rates among patients with PEA. In our study, ASY/PEA rhythm was significantly more common in victims of SCD versus survivors of SCA, thus contributing to previous studies. Even though the survival rate was high in our study, direct comparisons cannot be made with previous studies since we excluded SCA subjects with non-cardiac cause and rhythm recording delay of more than 30 min. Most efforts have been put into researching VF and VT, leaving ASY and PEA with less attention. The portion of VF/VT is decreasing and ASY and PEA increasing as presenting rhythms at the time of SCA. This is thought to be due to aging population and decreasing portion of acute coronary events among cases of SCA, while the portion of SCA due to nonischemic cardiac disease and non-cardiac etiology is increasing [1], [4], [5], [7], [8], [17]. In our earlier study, underlying nonischemic cardiac disease was associated with ASY/PEA rhythm at the time of SCA compared to ischemic cardiac disease [18].

While it is known that use of psychotropic medication and especially antipsychotics increases the risk of SCD [9], [10], [11], the effect of psychotropic medication on the initial rhythm at the time of SCA has not been largely studied. In our study, use of antipsychotics was an independent determinant of non-shockable rhythm at the time of SCA, which might partly explain the association between use of antipsychotics and SCD. The Oregon Sudden Unexpected Death Study demonstrated that use of antipsychotics and antidepressants increases the risk of PEA versus VF/VT at the time of SCA [7]. Their study did not include subjects with ASY as initial rhythm. Our results suggest that similar association extends to a larger group with non-shockable rhythm.

Antidepressants, especially tricyclic antidepressants, have also been reported to increase the risk of SCD [7], [11], [19]. Although, in a large cohort study by Leonard et al, out of 11 antidepressants, only mirtazapine had a statistically significant risk of ventricular arrhythmia and SCD [20]. In our study, use of antidepressants was not associated with non-shockable initial rhythm at the time of SCA. According to previous studies, benzodiazepines do not have any acknowledged proarrhythmic potential and have not been reported to increase the risk of SCD [19]. In our study, use of benzodiazepines was not independently associated with non-shockable initial rhythm at the time of SCA. Additionally, use of psychotropic medication was significantly more common in non-survivors compared to survivors. In a separate analysis, use of benzodiazepines, antidepressants, and antipsychotics was also significantly more common in non-survivors compared to survivors. However, use of psychotropic medication was not independently associated with death at the time of SCA when adjusted for delay and initial rhythm, implying the possibility that the risk of SCD in users of psychotropic medication might indeed be connected to the higher proportion of non-shockable rhythms.

While ASY and PEA are both non-shockable rhythms, they seem to occur partly through different mechanisms. At the time of SCA, VF/VT and PEA tend to eventually result in ASY if sinus rhythm is not recovered. Asystole may also occur as the initial rhythm, either due to a failure of the cardiac intrinsic electrical system or an extracardiac reason. A key mechanism for the occurrence of PEA is a contractile failure of the myocardium [21]. Most psychotropic medications increase the risk of an abnormally prolonged QT interval which can induce torsades de pointes and progress to VF [19]. Antipsychotics and antidepressants have also been reported to induce ion channel inhibition in the myocardium, which may result in arrhythmias and hypotension [22].

According to previous studies, psychotropic medication rarely lead to sudden death on a single QT-prolonging medication, but factors such as polypharmacy, recent initiation of a QT-prolonging medication, electrolyte abnormalities and preexisting arrhythmias increase the risk of SCD induced by psychotropics [23], [24]. However, psychotropic medication, especially antipsychotics, should only be prescribed to patients with a clear indication for psychotropic medication, such as a diagnosed psychiatric condition. Additionally, clinicians have to acknowledge that there are considerable cardiovascular comorbid factors among psychiatric patients using psychotropic medication [25]. Therefore, cardiovascular examination combined with electrocardiography and blood analyses might be necessary before prescribing a new psychotropic medication to a patient, especially if cardiovascular symptoms, such as chest pain, palpitations, dyspnea, or peripheral edema, are present.

Our study took into account all possible initial rhythms at the time of SCA. To our knowledge, the association between psychotropic medication and initial rhythm including ASY has not been studied before. Many researchers consider excluding cases of SCA with ASY as the initial rhythm to result in a more accurate approach due to the observation of ASY to often manifest as the end result of either VF/VT or PEA. In order to minimize the risk of VF/VT and PEA evolving into ASY, we excluded cases with an over 30-minute delay from collapse to recording of rhythm. In addition, the delay from collapse to recording of rhythm after exclusions was similar between the VF/VT group and the ASY/PEA group. Considering the significant proportion of ASY in SCA subjects, including all possible rhythms might offer a more comprehensive approach.

In previous studies, the cause of a sudden death has usually been estimated by prior medical history. In this study, all deaths were confirmed to be due to a cardiac cause by autopsy. We consider autopsy to be a more reliable way to determine the cause of a sudden death, for many sudden conditions, such as aortic dissection, pulmonary embolism and stroke, can lead to sudden collapse and might be determined as SCA due to a cardiac cause, if not autopsied. Furthermore, we adjusted the results of the study with the type of underlying cardiac disease. In our earlier study, underlying nonischemic cardiac disease was associated with ASY/PEA rhythm at the time of SCA compared to ischemic cardiac disease [18].

The statistical power of the study was limited due to the relatively small sample size. Because of this we did not examine ASY and PEA groups separately. As we analyzed different types of psychotropic medications separately, the sample sizes decreased considerably, limiting the reliability of the analysis. Also, neither dose nor duration of psychotropic medication use was evaluated in this study, and we did not know whether the patients were actually taking the medications, or if they were prescribed other medications not mentioned in the medical records of Oulu University Hospital. Several participants were users of multiple types of psychotropic medication, making it difficult to tell apart the effects of different medications. The use of psychotropic medication indicates the patients to have had a psychiatric condition, which makes it difficult to distinguish the effects of psychotropic medication and psychiatric disorder on the initial rhythm at the time of SCA. Unfortunately, we did not have more detailed information about the psychiatric conditions of the participants. The information on morbidity was limited, as history of diabetes was determined by use of metformin, and the body mass index was missing in a majority of the subjects. However, all causes of deaths were confirmed by autopsy.

Many previous studies have used categories of death and survival to hospital discharge, and as we did not possess information about further health conditions of the subjects who survived to hospital admission, comparing our results to previous SCA studies is complicated. Nevertheless, we consider the inclusion of the autopsy verified SCD group in our study a major strength, since most prior studies have excluded these subjects from their analyses and included only subjects who survived to hospital care. In our opinion, this poses a considerable bias in the previous studies towards survivors of SCA.

## 5 Conclusions

Use of psychotropic medication and especially antipsychotics is an independent determinant of a non-shockable rhythm and is thus associated with a lower survival rate at the time of SCA. This might partly explain the observation that use of psychotropic medication increases the risk of SCD. Use of antidepressants and benzodiazepines seem not to be an independent determinant of a non-shockable rhythm.

## CRediT authorship contribution statement

**Janna P. Kauppila:** Investigation, Writing - original draft, Formal analysis, Visualization. **Antti Hantula:** Investigation. **Lasse Pakanen:** Resources, Writing - review & editing. **Juha S. Perkiömäki:** Writing - review & editing, Supervision. **Matti Martikainen:** Resources. **Heikki V. Huikuri:** Conceptualization, Supervision, Writing - review & editing. **M. Juhani Junttila:** Conceptualization, Supervision, Project administration, Writing - review & editing.

## Declaration of Competing Interest

None.
